# Survey of professional views on sharing interim results by the Data Safety Monitoring Board (DSMB): what to share, with whom and why

**DOI:** 10.1186/s13063-018-2655-y

**Published:** 2018-05-21

**Authors:** Victoria Borg Debono, Lawrence Mbuagbaw, James Paul, Norman Buckley, Lehana Thabane

**Affiliations:** 1 Health Research Methods, Evidence and Impact, McMaster University, Hamilton, Canada; 2 Biostatistics Unit, St Joseph’s Healthcare, Hamilton, Canada; 3 Department of Anesthesia, McMaster University, Hamilton, Canada

**Keywords:** Data Safety Monitoring Board (DSMB), Data Monitoring Committee (DMC), Interim Result Sharing, Survey

## Abstract

**Background:**

Sharing interim results by the Data Safety Monitoring Board (DSMB) with non-DSMB members is an issue that can affect trial integrity. It is unclear what should be shared. This study assesses the views of professionals to understand what interim information should be shared at interim, with whom and why.

**Methods:**

Conducted an online survey of members of the Society of Clinical Trials (SCT) and International Society of Clinical Biostatistics (ISCB) in 2015 asking their professional views on sharing interim results. Email was used to advertise the survey and a link in the email was provided to the online survey.

**Results:**

Approximately 3136 (936 SCT members + 2200 ISCB members) members were invited. The response rate was 12% (371/3136). The majority reported the Interim Control Event Rate (IControlER) (149/237; 62.9% [95% CI, 56.7–69.0%]), Adaptive Conditional Power (ACP) (144/224; 64.3% [95% CI, 58.0%–70.6%]) and the Unconditional Conditional Power (UCP) (126/208; 60.6% [95% CI, 53.9–67.2%]) should not be shared with non-DSMB members. The majority reported that the Interim Combined Event Rate (ICombinedER) (168/262; 64.1% [95% CI, 58.0–69.9%]) should be shared with non-DSMB members particularly the steering committee (SC) because it does not unmask interim results and helps with monitoring trial progress, safety, and design assumptions.

**Conclusion:**

The IControlER and ACP are unmasking of interim results and should not be shared. The UCP is a technical measure that is potentially misleading and also should not be shared. The ICombinedER is usually known by the SC and sponsor making it easy to determine group rates if the IControlER is known. Though most respondents thought the ICombinedER should be shared with the SC as it does not unmask relative effects between groups, we do not recommend sharing the ICombinedER as it is flawed measure that can have multiple interpretations possibly suggesting that one group is performing better, worse or the same as a comparator group, leading to guesses about how groups are doing relative to one another.

**Electronic supplementary material:**

The online version of this article (10.1186/s13063-018-2655-y) contains supplementary material, which is available to authorized users.

## Background

Data Safety Monitoring Boards (DSMBs) are responsible for the stewardship of a trial [1, 2]. A trial can be negatively affected by the introduction of bias if the DSMB were to share interim trial results with non-DSMB members [1, 3, 4]. This is a serious concern for phase III trials that are usually used to provide definitive evidence on efficacy and safety outcomes [5, 6].

There is evidence in the academic literature to suggest that the issue with the DSMB sharing potentially unmasking interim results with non-DSMB members is prevalent [7]. Circumstances, where the DSMB may share potentially unmasking interim results, are when the DSMB makes a recommendation for early termination of a trial, the DSMB has concerns with the interim results given them for their scheduled interim review, the completion of the trial is endangered, the DSMB has a concern about the safety of trial participants, and there is a need to share interim results with a government regulator for early drug approval [7]. Other situations for sharing could be for adaptive confirmatory trials where interim results are needed to make planned study modifications, and when a trial has a long follow-up and some interim results may help a select patient group and their physician with a pivotal treatment choice. In these instances, unmasked interim results may be shared with non-DSMB members [7]. Four forms of interim results that could be shared that may be potentially unmasking are the Interim Combined Event Rate (ICombinedER), the Interim Control Event Rate (IControlER), the Adaptive Conditional Power (ACP) and the Unconditional Conditional Power (UCP). Definitions [8] of these interim result measures have been provided in Table 1. The ICombinedER, ACP and UCP provided at interim can be considered seemingly masked because they do not directly reveal the interim event rates for the trial’s treatment groups. However, the ICombinedER could indirectly reveal interim event rates per group if the control group event rate is known from the trial’s protocol or previous studies. Likewise, the ACP can reveal which group is doing relatively better. The IControlER, though revealing of the control group’s rate at interim, does not deliver information on how groups are doing relative to one another unless the ICombinedER is also given.

**Fig. 1:**
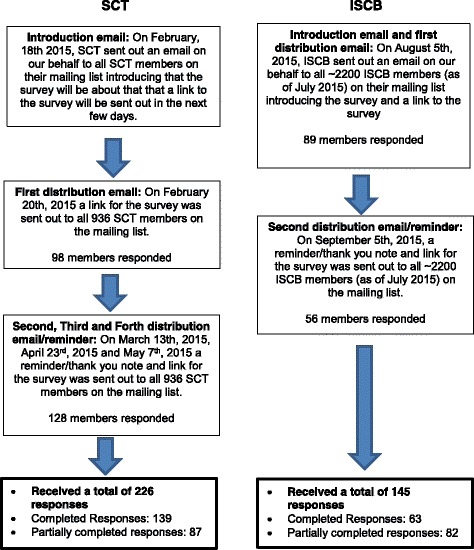
Flow diagram of the number of responses from the Society of Clinical Trials (SCT) and the International Society of Clinical Biostatistics (ISCB) after each reminder

**Table 1:** Definitions of four main forms of interim result measures

Interim Control Event Rate (IControlER)	The number of events observed among control participants at some planned interim point into the trial divided by number of control participants admitted at that same planned interim point (e.g., a planned interim point can be 6 months from the start of the trial)
	Example: • Total number of deaths in the placebo group, 6 months from the start of the trial = 15 • Total number of participants in the placebo group, 6 months from the start of the trial = 250 • Calculation: 15/250 = 0.06 or 6%
	Therefore, the Interim Control Event Rate at the trial’s interim analysis, 6 months from the start of the trial, is 6%
Interim Combined Event Rate (ICombinedER)	*“The total number of events observed at some planned interim point into the trial divided by the total number of participants admitted at that same planned interim point (e.g., a planned interim point can be 6 months from the start of the trial or after enrolling a certain number of participants)*
	Example: • *Total number of deaths in both the placebo group and new intervention group, 6 months from the start of the trial = 80* • *Total number of participants in both the placebo group and the new intervention group, 6 months from the start of the trial = 700* • *Calculation: 80/700 = 0.114 or 11.4%*
	*Therefore, the Interim Combined Event Rate at the trial’s interim analysis, 6 months from the start of the trial, is 11.4%.”* [8]
Adaptive Conditional Power (ACP)	*“The probability of rejecting the null hypothesis of no effect by the end of the trial (i.e., finding a statistically significant effect in favor of the intervention), at some predetermined interim point in the trial when the adaptive conditional power is scheduled to be calculated. The assumption made is that the observed interim effect (i.e., relative risk reduction) in the trial will remain the same until the end of the trial*
	*Example statement:* *Given the interim data (data collected 2 years into the trial that is planned to last for 3 years), and assuming the observed interim effect (i.e., relative risk reduction) at the 2-year point to be the true effect for the remainder of the trial, the probability of rejecting the null hypothesis of no effect (i.e., finding a statistically significant effect in favor of the intervention) at the end of the trial is 60%.*
	*The following pieces of information are used to calculate Adaptive Conditional Power at trial interim:* • *Control event rate and experimental event rate* • *Information Fraction; a ratio of the planned sample size and the number of patients recruited in the trial at the interim analysis* • *Z score and B value at interim* • *Drift parameter.”* [8]
Unconditional Conditional Power (UCP)	*“The probability of correctly rejecting the null hypothesis of no effect at the end of the trial (i.e., finding a statistically significant effect in favor of the intervention) and accepting the alternative hypothesis when indeed the alternative hypothesis is true, at some interim point in the trial* *The following pieces of information are used to calculate Unconditional Conditional Power at interim:* 1. *The hypothesized treatment effect at the design stage (i.e., relative risk reduction) of the trial, assuming the hypothesized treatment effect at the design stage to be true and correct for the remainder of the trial* 2. *The sample size calculated at the design stage for the trial* and 3. *The combined event rate calculated at the trial’s interim, assuming this rate to be true for the remainder of the trial*
	*Example statement:* *Given the interim combined event rate and assuming the treatment effect (i.e., relative risk reduction) hypothesized at the design stage of the trial to be true for the remainder of the trial, the probability of correctly rejecting the null hypothesis of no effect (i.e., finding a statistically significant effect in favor of the intervention) at the end of the trial is 89%.”* [8]

*DSMB* Data Safety Monitoring Board, *IControlER* Interim Control Event Rate, *ICombinedER* Interim Combined Event Rate, *ACP* Adaptive Conditional Power, *UCP* Unconditional Conditional Power

It is unclear whether these kinds of interim result measures should be shared, with whom and why. The objective of our survey was to collect empirical evidence and determine the professional opinions of those interested or involved in clinical trials on the issue of what interim information should be shared with non-DSMB members at interim and if so, with whom and under what circumstance. We will refer to the principal investigators (PIs), the steering committee (SC), sponsors, investigators, the independent unmasked statistician, the funder(s), or patients enrolled in the trial or any other party responsible for the conduct or completion of the trial as non-DSMB members and will be more specific when necessary.

## Methods

### Design of survey

We designed our survey to have 14 questions, many of which have parts to them (see Additional file 1 for all survey questions). Questions 1 to 6 solicit responses enabling a better understanding of what type of interim results or other types of interim information should be shared at interim, with whom and why. These first six questions had advanced branching such that latter parts of a particular question would appear depending on how the respondent answered an earlier part of the question (see Additional file 1). The definitions of the interim results were provided on the survey pages. We prioritized these questions first because they were vital to understanding what should be shared or not.

Questions 7 to 14 were demographic questions asking respondents about the roles they had in relation to trials, the number of trials they were involved with, their main profession by training, professional roles they have taken on, the kind of environment(s) they usually work in, the number of trials they were involved with that had some form of private industry sponsorship and the number of trials they have been involved with that had a DSMB.

#### Constructing and testing online survey

The online survey was constructed using fluidsurvey.com. We sent the first version of the online survey to 10 trial and health research experts at McMaster University, Hamilton, Ontario for pilot testing for content validity and clarity. They were asked for their feedback on the survey via email. We asked them specifically if they found the survey to be clear and the survey questions to be relevant to addressing our overall objective; to determine the professional opinions of those interested or involved in clinical trials on the issue of what interim information should be shared with non-DSMB members at interim. If something was not clear, we solicited their feedback to indicate where more clarity was needed and what they thought should be done to improve the survey. Nine out of 10 of the trial experts responded to the survey for pilot testing and feedback. We modified the online survey based on this feedback and created the final version of the online survey.

### Sampling

#### Target group and sampling

The target group for this survey was trialists or those involved in trials. We contacted two societies, the Society of Clinical Trials (SCT) and the International Society of Clinical Biostatistics (ISCB), and asked them for help in distributing our survey to their members. SCT members come from many sectors including industry, government, non-profit and advocacy groups, comprising professionals who are clinical investigators, biostatisticians, information technology specialists, project managers, clinical research associates and other professionals involved with the design, conduct and analysis of clinical trials [9]. ISCB members consist of clinicians, statisticians and members of other specialties including epidemiologists, clinical chemists and pharmacologists, who work or are interested in clinical biostatistics. Both of these societies agreed to distribute our survey [9, 10]. To get the best response rate possible, multiple emails were sent out to remind potential respondents of the survey. SCT sent out an initial email in February 2015 based on their own member mailing list (approximately 936 members around February 2015), letting potential respondents know about the online survey, its purpose, and the coming survey’s email invitation. The first survey email invitation was sent out February 2015 with a link to the online survey via fluidsurveys.com and, following the Dillman’s principles [11], a reminder email was sent out three more times (March 2015, April 2015 and May 2015) to encourage a good response. ISCB sent out the first survey email invitation in August 2015 based on their member mailing list (approximately 2200 current and past members around July 2015) with a link to the online survey via fluidsurveys.com [12]. A second reminder email was sent out in September 2015. In total, invitations for our survey were sent out to approximately 3136 (936 SCT members + 2200 ISCB members) members from both societies in total.

### Data collection and analysis

We used fluidsurveys.com to disseminate and collect responses. The software used to analyse the results was integrated software within fluidsurvey.com [12], WINPEPI Version 11.65 [13] and Microsoft Excel® 2010 [14]. Responses were collected anonymously. We report results in aggregate by count and percentages, indicating how many respondents chose a particular option and by mean where applicable with 95% confidence intervals (95% CIs). We summarized all written responses to questions qualitatively and quantitatively where applicable. For the questions related to whether the ICombinedER, IControlER, ACP or UCP should be shared, reasons for why an interim result measure should or should not be shared were assessed for emergent themes in relation to trial research. The description and the text given by respondents were first collated and then each response was read carefully. With iterative reading of the responses, similar reasons were grouped together. When no more groups of similar reasons existed, the groups that were there were then assessed for emerging overarching themes that were drawn from the reasons/responses within each group. This study was approved by the Hamilton Integrated Research Ethics Board (HiREB) approval [15].

## Results

We received 371 responses (202 complete responses, 169 partial or totally incomplete responses). Totally incomplete responses are participants who submitted a survey but did not answer any questions. Figure 1 summarizes the response rate (Fig. 1: Flow diagram of the number of responses from SCT and ISCB after each reminder). Best efforts were made to solicit as many responses as possible through multiple emails. Four reminder emails were sent in total (three to SCT and one to ISCB), as was allowed.

### Respondent demographics

Table 2 summarizes the main respondent demographics. The largest proportion of responses (42.0%) was from statisticians (156 out of 203 people responded to this question) and at least 53.6% of respondents were involved in at least one trial (203 responded to this question). About 50% (50.4%) of respondents were involved in at least one trial with DSMB monitoring (197 responded to this question) and the largest proportion of respondents (33.2%) usually work at a university or academic institution (202 responded to this question). Percentages are based on the total number of respondents to the survey (*n* = 371).

**Table 2:** Demographics of respondents (*n* = 371)

**Number of trials in which respondent has been involved** ^**a**^		**Number of trials the respondent has been involved with that had a Data and Safety Monitoring Board (DSMB) monitoring the trial** ^**b**^	
Number of trials	*n* (%)^q^	Number of trials	*n* (%)^q^
None 1 to 5 trials 6 to 10 trials 11 to 15 trials More than 15 trials Unknown ^A^	4 (1.1) 20 (5.4) 25 (6.7) 23 (6.2) 131 (35.3) 168 (45.3)	None 1 to 5 trials 6 to 10 trials 11 to 15 trials More than 15 trials Unknown^B^	10 (2.7) 34 (9.2) 37 (10.0) 28 (7.5) 88 (23.7) 174 (46.9)
**Number of trials the respondent has been involved with that had some form of private industry sponsorship** ^**c**^		**Primary profession by training** ^**d**^	
Number of trials	*n* (%)^q^	Main profession	*n* (%)^q^
None 1 to 5 trials 6 to 10 trials 11 to 15 trials More than 15 trials Unknown^C^	29 (7.8) 69 (18.6) 25 (6.7) 14 (3.8) 64 (17.3) 170 (45.8)	Mathematician/statistician/biostatistician Methodological scientist/research methodologist Physician Epidemiologist Research or clinical trial coordinator Ethics specialist Trialist Analyst Computer programmer Trial manager Trial monitor Unknown^D^	156 (42.0) 21 (5.7) 10 (2.7) 5 (1.3) 3 (0.8) 2 (0.5) 2 (0.5) 1 (0.3) 1 (0.3) 1 (0.3) 1 (0.3) 168 (45.3)
**Usual work setting of respondents** ^**e**^		**Other work settings of respondents** ^f *^	
Place of work	*n* (%)^q^	Other places of work	*n* (%)^q^
University or academic institution Private or contracted research company Pharmaceutical company Government research group Hospital Government regulatory body Academic university hospital Medical device company Private practice Retired Unknown^E^	123 (33.2) 28 (7.5) 17 (4.6) 13 (3.5) 10 (2.7) 5 (1.3) 3 (0.8) 1 (0.3) 1 (0.3) 1 (0.3) 169 (46.0)	Hospital University or academic institution Pharmaceutical company Private or contracted research company Government research group Medical or health clinic Government regulatory body Medical device company Private practice Consulting entity Data Safety Monitoring Board Health maintenance organization (research department) Unknown^F^	36 (9.7) 35 (9.4) 18 (4.9) 15 (4.0) 15 (4.0) 12 (3.2) 11 (3.0) 7 (1.9) 4 (1.1) 1 (0.3) 1 (0.3) 1 (0.3) 268 (72.2)
**Roles respondents have taken on in relation to trial operation** ^g*^		**Professional roles respondents have taken on** ^h*^	
Roles in relation to the trial	*n* (%)^q^	Professional roles	*n* (%)^q^
Trial statistician Data Safety Monitoring Board member Trialist or investigator (i.e., co-investigator in a trial) Data analyst Principal investigator (PI) of a clinical trial Sponsor representative Funder representative Data manager Steering Committee Independent unblinded reporting Statistician to the DSMB Trial coordinator Data coordinator/manager Government regulator Consultant Unknown ^G^	161 (43.4) 136 (36.7) 88 (23.7) 68 (18.3) 30 (8.1) 26 (7.0) 18 (4.9) 11 (3.0) 11 (3.0) 9 (2.4) 7 (1.9) 4 (1.1) 3 (0.8) 3 (0.8) 171 (46.1)	Methodological scientist/research methodologist Epidemiologist Mathematician/statistician Data manager Computer programmer Research or clinical trial coordinator Computer scientist Ethics specialist Physician Information technologist Lawyer Medical laboratory technician Medical laboratory scientist Nurse or nurse practitioner Biochemist Engineer Regulator Teacher Therapist Trial management Unknown^H^	89 (24.0) 63 (17.0) 48 (12.9) 36 (9.7) 35 (9.4) 12 (3.2) 10 (2.7) 10 (2.7) 9 (2.4) 6 (1.6) 2 (0.5) 1 (0.3) 1 (0.3) 1 (0.3) 1 (0.3) 1 (0.3) 1 (0.3) 1 (0.3) 1 (0.3) 1 (0.3) 211 (56.9)

^a^Based on Survey Question 8. Total of 203 responses to this question; ^A^ Unknown because 168 respondents did not answer this question. ^b^ Based on Survey Question 14. Total of 197 responses to this question; ^B^ Unknown because 174 respondents did not answer this question. ^c^ Based on Survey Question 13. Total of 201 responses to this question; ^C^ Unknown because 170 respondents did not answer this question. ^d^ Based on Survey Question 9. Total of 203 responses to this question; ^D^ Unknown because 168 respondents did not answer this question. ^e^ Based on Survey Question 11. Total of 202 responses to this question; ^E^ Unknown because 169 respondents did not answer this question. ^f^ Based on Survey Question 14. Total of 197 responses to this question. Respondents to this question were asked to select all roles (or categories) that applied to them; thus, a respondent can be in more than one category; ^F^ Unknown because 268 respondents did not answer this question. ^g^ Based on Survey Question 7. Total of 200 responses to this question. Respondents to this question were asked to select all roles (or categories) that applied to them; thus, a respondent can be in more than one category; ^G^ Unknown because 171 respondents did not answer this question. ^h^ Based on Survey Question 10. Total of 160 responses to this question. Respondents to this question were asked to select all roles (or categories) that applied to them; thus, a respondent can be in more than one category; ^H^ Unknown because 211 respondents did not answer this question. ^q^ Percentages based on the 371 total respondents to this survey

*Respondents could have selected more than one option; thus, it is possible that the percentages add up to more than 100%

### Main results for questions 1 to 4

Table 3 summarizes the main results regarding sharing the ICombinedER, IControlER, ACP and UCP, respectively.

**Table 3:** Summary of Results for Sharing Certain Interim Results

***Interim Combined Event Rate***	
1 a) During an ongoing Randomized Controlled Trial (RCT), do you think that the Data Safety Monitoring Board (DSMB) for an RCT should share the Interim Combined Event Rate with ANY of the following parties?	
Response	Results (*n*; % [95% CI]), *N* = 262
Yes	168; 64.1% [58.0% to 69.9%];
With whom?*	
B. The Steering Committee A. The Sponsor C. The Investigator(s) D. The Funder(s) E. Other, Please Specify: • Institutional Review Board or Research Ethics Boards, • Regulatory Bodies, • Blinded Statistician on Steering Committee, • Study Statistician • Participants • Professional public	142; 54.2% [48.2% to 60.2%]; 101; 38.5% [32.7% to 44.4%]; 80; 30.5% [25.0% to 36.1%]; 64; 24.4% [19.2% to 29.6%]; 15; 5.7% [2.9% to 8.5%];
No (F. None of the Above)	94; 35.9% [30.1% to 41.7%]
1 b) How useful is it to share the Interim Combined Event Rates at interim? (On a scale from 0 to 10 where 0 is Not Useful at All and 10 is Very Useful) Question 1 b. answered only by those who answered A, B, C, D or E to Question 1 a.	
Results (Mean [95% CI]; Median [IQR]), *N* = 146	
6.97 [6.62 to 7.31]; 7 [6-8]	
***Interim Control Event Rate***	
2 a) During an ongoing Randomized Controlled Trial (RCT), do you think that the Data Safety Monitoring Board (DSMB) for an RCT should share the Interim Control Event Rate with ANY of the following parties?	
Response	Results (*n*; % [95% CI]), *N* = 237
Yes	88; 37.1% [31.0% to 43.3%]
With whom?*	
B. The Steering Committee C. The Investigator(s) A. The Sponsor D. The Funder(s) E. Other, Please Specify: • Institutional Review Board or Research Ethics Boards, • Regulatory Bodies • Professional Public • Study Statistician	60; 25.3% [19.8% to 30.9%] 35; 14.8% [10.3% to 19.3%] 33; 13.9% [9.5% to 18.3%] 30; 12.7% [8.4% to 16.9%] 22; 9.3% [5.6% to 13.0%]
No (F. None of the Above)	149; 62.9% [56.7% to 69.0%]
2 b) How useful is it to share the Interim Control Event Rates at interim? (On a scale from 0 to 10 where 0 is Not Useful at All and 10 is Very Useful) Question 2 b. answered only by those who answered A, B, C, D or E to Question 2 a.	
Results (Mean [95% CI]; Median [IQR]), *N* = 72	
7.03 [6.55 to 7.50]; 7 [5-8]	
***Adaptive Conditional Power***	
3 a) During an ongoing Randomized Controlled Trial (RCT), do you think that the Data Safety Monitoring Board (DSMB) for an RCT should share the Adaptive Conditional Power with ANY of the following parties	
Response	Results (*n*; % [95% CI]), *N* = 224
Yes	80; 35.7% [29.4% to 42.0%]
With whom?*	
B. The Steering Committee A. The Sponsor C. The Investigator(s) D. The Funder(s) E. Other, Please Specify • Trial statistician, • Pre-specified members of the sponsor and steering committee, • Professional public, • Institutional Review Board or Research Ethics Boards	45; 20.1% [14.8% to 25.3%] 34; 15.2% [10.5% to 19.9%] 27; 12.1% [7.8% to 16.3%] 22; 9.8% [5.9% to 13.7%] 21; 9.4% [5.6% to 13.2%]
No (F. None of the Above)	144; 64.3% [58.0% to 70.6%]
3 b) How useful is it to share the Adaptive Conditional Power at interim? (On a scale from 0 to 10 where 0 is Not Useful at All and 10 is Very Useful) Question 3 b. answered only by those who answered A, B, C, D or E to Question 3 a.	
Results (Mean [95% CI]; Median [IQR]), *N* = 66	
6.64 [6.08 to 7.20]; 7 [5-8]	
***Unconditional Conditional Power***	
4 a) During an ongoing Randomized Controlled Trial (RCT), do you think that the Data Safety Monitoring Board (DSMB) for an RCT should share the Unconditional Conditional Power with ANY of the following parties?	
Response	Results (*n*; % [95% CI]), *N* = 208
Yes	82; 39.4% [32.8% to 46.1%]
With whom?*	
B. The Steering Committee A. The Sponsor C. The Investigator(s) D. The Funder(s)* E. Other, Please Specify • Pre-specified with whom such as selected members of the sponsor or funder who do not see patients • Study statistician • Steering committee • Professional public • Institutional Review Board or Research Ethics Boards	57; 27.4% [21.3% to 33.5%] 42; 20.2% [14.7% to 25.6%] 30; 14.4% [9.6% to 19.2%] 29; 13.9% [9.2% to 18.6%] 17; 8.2% [4.4% to 11.9%]
No (F. None of the Above)	126; 60.6% [53.9% to 67.2%]
4 b) How useful is it to share the Unconditional Conditional Power at interim? (On a scale from 0 to 10 where 0 is Not Useful at All and 10 is Very Useful) Question 4 b. answered only by those who answered A, B, C, D or E to Question 4 a	
Results (Mean [95% CI]; Median [IQR]), *N* = 67	
6.64 [6.08 to 7.20]; 7 [5-8]	

IQR (Interquartile Range)

* Respondents could have selected more than one option thus it is possible that the percentages add up to more than 100%.

#### Interim Combined Event Rate (ICombinedER)

The majority of respondents (168/262; 64.1% [95% CI, 58.0% to 69.9%]) reported that the ICombinedER should be shared. The majority of those who said that it should be shared reported that it should be shared with the SC (142/262; 54.2% [95% CI, 48.2% to 60.2%]). For those that said that the ICombinedER should be shared, we then asked how useful it was to share the ICombinedER and those that responded gave it a mean score of 6.97 (95% CI, 6.62 to 7.31) and a median score of 7 (interquartile range (IQR) 6–8), on a scale from 0 to 10 where 0 is Not Useful at All and 10 is Very Useful. Also, for those who said that the ICombinedER should be shared (yes), we asked those respondents to briefly explain why they thought the ICombinedERs should be shared by the DSMB at interim (*n* = 131/168). One theme emerged from their responses as to why the ICombinedERs should be shared; it is unlikely to threaten the integrity of the trial. A summary of details said by respondents related to this theme are as follows. Firstly, sharing the ICombinedER is unlikely to threaten the integrity of the trial as it does not tell you anything about the effect size between groups and allows investigators, sponsors and the SC to be informed about trial progress, check design assumptions and make appropriate corrective adaptations to protect the trial’s integrity and participant/patient safety. And secondly, most sponsors and SCs would be able to calculate the ICombinedER because they would already have access to the pooled database.

An additional point made as a word of caution was that either the ICombinedER or the IControlER should be shared, not both, as it would be unmasking of how the intervention group is doing. If the control event rate is predictable from the academic literature, then the ICombinedER should not be shared. The default is to not share unless pre-specified with whom and when. Being given the ICombinedER also does not stop guesses about effect sizes to be made by non-DSMB members.

#### Interim Control Event Rate (IControlER)

The majority of respondents (149/237; 62.9% [95% CI, 56.7% to 69.0%]) reported that the IControlER should not be shared with anyone. These respondents were then asked briefly to explain why the IControlER should not be shared with anyone at interim (*n* = 120/149). One theme emerged from their responses as to why the IControlER should not be shared; the IControlER is unnecessary to share, misleading and potentially unmasking of interim effects between groups. A summary of details said by respondents related to this theme is as follows. Firstly, the IControlER may be unmasking of interim results as the SC or the sponsor usually has access to pooled data and being given this would allow them to back calculate the interim rate in the intervention group, thus jeopardizing the integrity of the trial. Secondly, the IControlER is an unreliable estimate at interim and there is no reason or need to share the IControlER since the DSMB can make necessary recommendations to the SC if needed to protect the integrity of the trial and non-DSMB members need to trust the DSMB on that task. And lastly, the SC or the sponsor needing to know the IControlER would have to outweigh the potential threat to trial integrity and validity because of the potential to introduce trial bias.

#### Adaptive Conditional Power (ACP)

The majority of respondents (144/224; 64.3% [95% CI, 58.0% to 70.6%]) reported that the ACP should not be shared with anyone. These respondents were then asked to briefly explain why the ACP should not be shared with anyone or any party at a trial’s interim (*n* = 117/144). Two themes emerged from their responses as to why the ACP should not be shared: 1) The ACP is potentially unmasking of interim results and 2) The ACP is a highly technical measure to interpret. A summary of details said by respondents related to these themes are as follows. Firstly, the ACP is very informative of the presence or absence of relative treatment effects and hence it is partially unmasking to those responsible for the trial’s conduct and it is unlikely that the ACP will remain confidential if shared. Secondly, the ACP gives non-DSMB members an opportunity to do a back calculation for the treatment effect potentially biasing the trial should the behavior of the trial stakeholders and those responsible for trial conduct be modified from knowing such information. And lastly, funders are typically not qualified to assess the relevance of such information.

#### Unconditional Conditional Power (UCP)

The majority of respondents (126/208; 60.6% [95% CI, 53.9% to 67.2%]) reported that the UCP should not be shared with anyone. These respondents were then asked to briefly explain why the UCP should not be shared with anyone at a trial’s interim (*n* = 96/126). One theme emerged from their responses as to why the UCP should not be shared; the UCP is a technical measure that is potentially misleading. A summary of details said by respondents related to this theme is as follows. There is confusion around what this measure exactly indicates so much so that it could be interpreted incorrectly as an adaptive conditional power and hence releasing this information will result in speculation, most often incorrect, about the components that are used to generate the UCP which could influence behavior at all levels of study conduct. Secondly, the UCP is not useful information and has questionable utility.

### Sharing other kinds of information

About half of the respondents to the question about sharing other kinds of information at interim by the DSMB with non-DSMB members reported that no other information should be shared (109/210; 51.9% [95% CI, 45.0% to 58.8%]) while 101 out of 210 (48.1% [95% CI, 41.2% to 55.0%]) respondents said yes, that other information should be shared. Table 4 summarizes all the responses. The top three responses for those that said “yes” to share other information at interim by the DSMB with non-DSMB were information about trial conduct (67; 31.9% [95% CI, 25.7% to 38.6%]), a safety issue or concern (50; 23.8% [95% CI, 18.3% to 30.1%]) and DSMB interim trial recommendations (21; 10.0% (95% CI, 6.3% to 14.9%)). The mean usefulness to share these three types of interim information were 9.16 (95% CI, 8.89 to 9.42), 9.35 (95% CI, 9.02 to 9.69) and 9.52 (95% CI, 9.08 to 9.96), respectively. Additionally, the medians for the usefulness to share these three types of interim information were 10 (IQR, 8–10), 10 (IQR, 9–10) and 10 (IQR, 10-10) [10], respectively. With whom to share varied. For information about trial conduct, it was indicated that this should be shared with the sponsor, SC, investigators or any relevant party. For information about a safety issue or concern, it was reported that this should be shared with the sponsor, SC, investigators or the Ethics Committee. For DSMB interim trial recommendation, it was indicated that this should be shared with the sponsor or the SC.

**Table 4:** Other information that should be shared

**Do you think that any other information should be shared during the interim of a randomized controlled trial by the Data Safety Monitoring Board (DSMB)?**				
Total responses to question: 210				
**Response**	**Count; % [95% CI]**			
No	109; 51.9% [45.0% to 58.8%]			
Yes	101; 48.1% [41.2% to 55.0%]			
**For those that answered Yes, what other information the DSMB should share at a trial’s interim, with whom it should be shared, why, and how useful it is to share that information?**				
**What should be shared?**	**Count; % [95% CI]** ^**A**^	**With whom should that information be shared?**	**Why should this information be shared?**	Usefulness to share* **mean [95% CI];** **median [IQR]**
Information about trial conduct (e.g., protocol adherence, operational issues, enrollment, recruitment, treatment adherence, trial management, data quality and completeness)	67; 31.9% [25.7% to 38.6%]	• Sponsor, • Steering Committee, • Investigators, or any relevant party	To ensure that the trial is conducted well with integrity and ethically. Information about trial conduct issues will help instigate corrective measures	9.16 [8.89 to 9.42]; 10 [8–10]
Safety Issue or concern	50; 23.8% [18.3% to 30.1%]	• Sponsor • Steering Committee • Investigator(s) • Ethics Committee	Based on the type of safety concern, investigators may need to increase monitoring to protect patient safety, change the trial’s protocol or request new consent from enrolled patients based on new safety risk	9.35 [9.02 to 9.69]; 10 [9–10]
DSMB trial recommendations such as stopping or continuing the trial and possible sample size adjustment. Information shared does not include unmasking group information.	21; 10.0% [6.3% to 14.9%]	• Sponsor • Steering Committee	To protect the trial’s integrity, patient safety, and trial resources. Due diligence to patients and the public good	9.52 [9.08 to 9.96]; 10 [10–10]
Overall patient baseline characteristics	9; 4.3% [2.0% to 8.0%]	• Any relevant party	Help study team understand if their enrollment is targeting the intended population. Protect the generalizability of the study. Help evaluate recruitment procedures and analysis plan	8.0 [7.27 to 8.73]; 8 [8–8]
Any relevant data or raw data	4; 1.9% [0.5% to 4.8%]	• Any relevant party	Sharing allows for broader stakeholder discussion of the benefits of treatment versus the risks of adverse events than just a committee with minimum involvement. There is no harm in this if efficacy stopping rules are pre-specified	9.33 [8.68 to 9.99]; 9 [9–9.5]
Important information from outside of the trial that is relevant to the current trial, the enrolled patients, the sponsor and the investigators	2; 1.0% [0.1% to 3.4%]	• Steering Committee • Study team members	During a long-term trial, results from other trials may affect the ethics, scientific rationale, care of patients and conduct of the current trial	9.5 [8.52 to 10]; 9.5 [9.25–9.75]

^A^Respondents could have indicated more than one of other type of item; thus, it is possible that the percentages add up to more than 100%

*On a scale between 0 to 10 (where 0 is Not Useful at All and 10 is Very Useful)

*IQR* interquartile range, *CI* confidence interval

### Sharing of interim information as indicated in encountered DSMB charters by respondents

About half (104/207; 50.2% [95% CI, 43.3 to 57.2%]) of the respondents to this question about sharing interim information as indicated by the DSMB charters they encountered reported “yes,” that they were involved in a trial where sharing such information was explicitly stated in the DSMB charter. Table 5 summarizes all the responses. For those that said “yes” to the first part of this question, they were then asked which of the following pieces of interim information (ICombinedER, IControlER, ACP and UCP) should be shared during the interim of a trial, with whom and under what circumstance the sharing would happen according to the DSMB charters they encountered as well as any other additional information. The greatest proportion of respondents (55/207, 26.6% [95% CI, 20.7 to 33.1%]) reported that the ICombinedER would be shared with various parties if the overall rate was much lower than hypothesized, if there was a need to adjust the sample size or re-assess the trial’s power, if benchmarks were not met, if there was a safety or ethical issue, or if there was a recommendation from the DSMB to stop the trial because of futility or efficacy. Various respondents reported that the ICombinedER would be shared with the sponsor, investigator, funder, SC, regulator or another relevant party when deemed appropriate.

**Table 5:** Sharing of interim information indicated in encountered Data Safety Monitoring Board (DSMB) charters

**Have you ever been involved in a trial where it was explicitly stated in the Data Safety Monitoring Board (DSMB) charter** ***what interim information/data/results should be shared*** **and** ***with whom that information should be shared*** **during the trial’s interim?**			
Total responses to question: 207			
**Response**	**Count; % [95% CI]**		
No	103; 49.8% [42.8% to 56.7%]		
Yes	104; 50.2% [43.3% to 57.2%]		
**For those that answered “yes” and according to any DSMB charter(s) they encountered, which of the following pieces of interim information** ***should be shared*** **during the interim of a trial,** ***with whom*** **and** ***under what circumstance*** **the sharing would happen.**			
**Interim Information**	**Count; % [95% CI]** ^**B**^	**With whom should that information be shared?**	**Under what circumstance this information should be shared according to the charter? Summary of responses**
Interim Combined Event Rate	55; 26.6% [20.7% to 33.1%]	Various parties indicated: • Sponsor • Investigator • Funder • Steering Committee • Regulator • Other relevant parties	Various responses were given. Singular parties: • With the investigator: if the overall rate was much lower than hypothesized and if there was a need to adjust the sample size • With the Steering Committee: always shared at each meeting without restrictions. To help with potential sample size re-estimation and re-assess power without unmasking group event rates. When the overall rate is much lower than anticipated • With the sponsor: shared during open session report. To help with potential sample size re-estimation. Help sponsor anticipate the length of the trial. Sharing was up to the DSMB’s discretion • With the regulatory agency: If there was a safety issue A combination of parties: • With select members of the sponsor, steering committee or investigator(s): pre-specified in the charter. When benchmarks are not met or when there is determined need for a sample size re-estimation. Need to share if there was a recommendation from the DSMB to stop the trial because of futility or efficacy. Such information is only used for internal decision-making and is not for publication or further dissemination • With the sponsor, funder or investigator(s): once accrual was complete and the primary outcome was known for at least a certain set percentage of those enrolled. It was also indicated that this information was shared at every planned interim look • With relevant parties: for safety and ethical issues
Interim Control Event Rate	16; 7.7% [4.5% to 12.2%]	Various parties indicated: • Sponsor • Investigator • Funder • Steering Committee • Regulator • Other relevant parties	Various responses were given. Singular parties: • With the Steering Committee: pre-specified in the charter. If the event rate was different from what was pre-specified in the protocol • With the sponsor: when there is a futility analysis and if the interim control event rate differed majorly from the design assumptions • With the regulatory agency: if there was a safety issue A combination of parties: • Select members of sponsor/funder, Steering Committee or investigator(s): o Pre-specified in the charter. sharing this information was not data driven o It would be shared once accrual was complete and the primary outcome was known for at least a certain set percentage of those enrolled. It was also indicated in another instance that interim control event rate was shared at every planned interim look • With relevant parties: For safety and ethical issues
Adaptive Conditional Power	19; 9.2% [5.6% to 13.9%]	Various parties indicated: • Sponsor • Investigator • Funder • Steering Committee • Other relevant parties	Various responses were given. Singular parties: • With the sponsor: would be shared at interim at the time of formal futility analysis • With the Steering Committee: would be shared at interim at the time of formal futility analysis and when a boundary was crossed. Also shared when there was a need for a management decision to be made A combination of parties: • Select members of sponsor/funder, Steering Committee or investigator(s): if the adaptive conditional power falls below a pre-fixed level or when there was data supporting stopping the trial. Pre-specified in the charter. Such information is only used for internal decision-making and is not for publication or further dissemination. In one instance it was also shared at the annual meeting report • With relevant parties: for safety and ethical issues
Unconditional Conditional Power	18; 8.7% [5.3% to 13.4%]	Various parties indicated: • Sponsor • Investigator • Funder • Steering Committee • Regulator • Other relevant parties	Various responses were given. Singular parties: • With the sponsor: would be shared at interim at the time of formal futility analysis and for a needed sample size recalculation • With the Steering Committee: would be shared at interim when there was a clear benefit or harm to whatever was being investigated and to re-assess power without unmasking interim results A combination of parties: • With select members of sponsor/funder, Steering Committee or investigator(s): pre-specified in the charter. When there was data supporting stopping the trial • With relevant parties: for safety and ethical issues There was an argument that such information is implicitly available, even if it is not directly provided
Other information			
Information about trial conduct	21; 10.1% [6.4 to 15.1%]	Various parties indicated: • Sponsor • Investigator • Funder • Steering Committee • Regulator	A combination of parties: • With the sponsor/funder, Steering Committee, investigator(s) or regulator if needed: This is not confidential information and should be shared during DSMB open sessions according to the charter with any relevant party at the open session and those responsible for the conduct of the trial to ensure the integrity of the trial’s conduct and correct problems as soon as possible
Safety issue or concern	16; 7.7% [4.5% to 12.2%]	Various parties indicated: • Sponsor • Investigator • Funder • Steering Committee • Regulator	A combination of parties: • With the sponsor/funder, Steering Committee, investigator(s) or regulator if needed: This is not confidential information and should be shared during DSMB open sessions according to the charter with any relevant party at the open session and those responsible for the conduct of the trial. It is important to share this information to help those responsible for the trial’s conduct to ensure participant safety
DSMB trial recommendations such as stopping or continuing the trial and possible sample size adjustment.	15; 7.2% [4.1% to 11.7%]	Various parties indicated: • Sponsor • Steering Committee	A combination of parties: • With the Steering Committee or sponsor: Pre-specified in the charter. Typical information shared in this circumstance would not include unmasked group information. However, it was indicated that if there cases where unmasked information would be shared if the decision to stop the trial has been made (e.g., for futility, efficacy or if some other pre-specified boundary has been reached)
Overall patient baseline characteristics	3; 1.4% [0.3% to 4.2%]	Various parties indicated: • Sponsor • Investigator • Funder • Steering Committee • Regulator	A combination of parties: • Sponsor/funder, Steering Committee, investigator(s) or regulator if needed: this is not confidential information would be shared during DSMB open sessions according to the charter with any relevant party at the open session and those responsible for the conduct of the trial
Unmasked treatment arm information	1; 0.5% [0.01% to 2.7%]	Various parties indicated: • Sponsor • Investigator • Public	A combination of parties: • With the sponsor, investigator(s) or public: it was also mentioned that primary outcome data by treatment group was once shared with the sponsor, investigator or public if the primary outcome is known for at least set percentage of trial patients and the target sample size was enrolled

Legend: *CI* confidence interval, *DSMB* Data Safety Monitoring Board

^B^Respondents could have indicated more than one item; thus, it is possible that the percentages add up to more than 100%

## Discussion

### Key findings

Our results empirically show that the ICombinedER is the only interim result measure where the majority of respondents think that the DSMB for a randomized controlled trial (RCT) should share with non-DSMB members. The majority of respondents indicate that it could be shared specifically with the SC. However, they did not give the ICombinedER a very high score on usefulness, only a moderate score of 6.97. Their reasoning generally for sharing this measure is that it does not tell you anything about relative effects between compared groups in a trial so it does not do any harm in terms of unmasking interim results and keeps investigators informed about the trial’s progress. They do indicate though that guesses can be made and that the ICombinedER should not be shared if the IControlER is known as having both can be unmasking of relative effects. The reason we think this measure was rated in the moderate range (6.97) in terms of usefulness is that many needed changes or decisions about the trial based on the ICombinedER can be suggested by the DSMB from their own interim review without the need to necessarily share the ICombinedER. The minority of respondents who said you do not need to share the ICombinedER indicated that the DSMB can recommend the needed changes or adaptations to the trial to the SC or sponsor without having to release the ICombinedER to them. The usefulness in sharing this measure is questionable if the DSMB is entrusted to guide the SC to make needed changes and decisions based on the DSMB’s review of the interim data. Even though the majority of respondents indicated that the ICombinedER should be shared, we do not recommend sharing it. Part of the reason for not sharing was indicated by respondents in that guesses can be made about comparative effects between treatment groups at interim. There is evidence to suggest in the academic literature [8] that the ICombinedER is a flawed measure to share and rely on as it can be compatible with any of three types of interim results: 1) one group (e.g., drug X) is performing better than another (e.g., placebo), 2) one group is doing worse than another or 3) both groups are performing the same. For instance, in this scenario question based survey [8], respondents correctly pointed out that having been given the ICombinedER of 0.34 or 34% for mortality in a hypothetical interim trial scenario could mean a 25% relative risk reduction, 25% relative risk increase or about a 2% relative risk reduction (where both groups are performing about the same). This flaw in sharing this measure is also dangerous as non-DSMB members could make speculations about comparative effects between treatment groups at interim that could consciously or subconsciously alter their behavior towards the trial, introducing bias.

Our results also empirically show that IControlER, the ACP and UCP are measures where the majority of respondents think that the DSMB for an RCT should not share with any non-DSMB member. Their reasoning generally for not sharing the IControlER and the ACP is that it can be unmasking of interim results, hence jeopardizing the integrity of the trial and potentially introducing bias. The IControlER can be directly unmasking because in many cases, the SC or the sponsor has access to pooled data, and being given the IControlER would allow them to back calculate the event rate for the intervention group. The ACP is very informative of the presence or absence of a relative treatment effect and hence it is partially unmasking to those responsible for the trial’s conduct. It was indicated that everyone involved in the trial should remain unaware of such interim results so that they can carry on enrolling, treating and following up with patients without being influenced by speculations and knowledge that could cause the introduction of trial bias. As for the UCP, comments against sharing correctly pointed out there is the potential for misunderstanding this result measure as it is simply computed under the original alternative hypothesis, not the current interim group event rates. Releasing this information could result in speculation of relative effects between groups, most often incorrect, possibly influencing behavior at all levels of study conduct. We think that the majority of respondents are correct when they say that these latter three measures should not be shared with any non-DSMB members because of the potential to introduce bias in the trial from having such information. We believe the ACP to be informative of relative group effects and the UPC to be a confusing measure that is misunderstood and possibly misinterpreted as an ACP as suggested by evidence [8]. If these types of interim results are to be shared by the DSMB, they should be shared with the SC at times when trial futility is in question or there is a major safety concern and such situations should be pre-specified in the protocol or DSMB charter. Otherwise, it seems best to let the DSMB be stewards of the trial. Respondents to our survey realize that there is a lot of risk to the integrity of the trial when sharing the latter three measures with non-DSMB members.

Beyond these four interim results, respondents indicated sharing other types of information that is typical of what is usually shared in practice. This included information on trial conduct, a safety issue or concern, DSMB interim recommendations, overall patient baseline characteristics and important information from outside the trial; all of which is very useful information that helps those responsible for the study to protect trial integrity and safety. This type of information also scored high by respondents on its usefulness for sharing at interim by the DSMB because such information provides the SC and the sponsor information needed to ensure good trial conduct without needing to unmask any group comparative results on the outcomes of interest. Sharing this type of information, when and for what reason, should be determined and agreed upon by the SC and the DSMB a priori and stated within the trial protocol and DSMB charter.

We also found out that about half of the respondents were involved in a trial where sharing interim results was explicitly stated in the DSMB charter and with whom that information should be shared during the trial’s interim. It is reassuring that there has been consideration given by trialists, before the commencement of a trial, about the possible need to share certain types of interim results with non-DSMB members and that such a need to share is explicitly stated a priori. However, this is not enough. We recommend that all trials should consider situations when there may be a need for the DSMB to share certain types of interim information and with whom. It needs to be explicit in DSMB charters how those situations that may entail sharing interim results will be handled to minimize trial bias.

### Findings compared to similar studies

This study is unique in that it empirically evaluates and focuses on whether four main forms of interim results should be shared, with whom, the usefulness of sharing that result measure, and why it should be shared, by soliciting the views of those involved in trials. A scenario-based survey published in 2017 asked trial experts how they interpreted the ICombinedER, ACP and UCP when shared in a hypothetical trial scenario. They concluded from their results that knowledge of these three interim measures should not be shared by DSMB with non-DSMB members at interim as they may mislead or unmask interim results, potentially introducing trial bias [8]. This previous survey corroborates our findings in that the majority of trial experts who responded to our survey also think the ACP and UPC should not be shared with non-DSMB members. However, the majority of respondents from our survey thought that the ICombinedER should be shared because it is not directly unmasking of the event rate per group and keeps investigators informed about the trial’s progress. They also indicated in this survey that guesses can be made about the effects between treatment group with this information; thus, caution and protocol pre-specification should be exercised when sharing this kind of information.

Six other surveys found, dating from 1999 to 2011, did not specifically focus on the issue of the DSMB sharing interim results, and were very limited in regards to the amount of detail they collected regarding what should be shared by the DSMB, with whom and why. These surveys globally looked at data monitoring practices and so each one does not provide a complete picture of the issue of the DSMB sharing interim results with non-DSMB members even when assessed as a group of articles. In general, we did find that three of the six surveys found [16–18] (one qualitatively and two quantitatively reported) support the view that interim results should not be shared by the DSMB with at least one type of non-DSMB member. Two surveys [17, 19] (one qualitatively and one quantitatively reported) showed support for the view that interim results should be shared by the DSMB with at least one type of non-DSMB member. One survey [17] (quantitatively reported) supports that interim results should be not be shared except in particular circumstances.

### Key limitations

In regards to limitations of our study, we had a very low response rate despite best efforts to solicit responses. We do not have any way of knowing how our non-respondents were different from our respondents. We do know from our demographic information that the largest proportion of our respondents were statisticians and about half were involved in at least one trial which reassured us that many of our respondents were most likely familiar with calculated interim result measures in a trial. Another limitation of our survey is that there was a lot of missing data. Though we received 371 responses, 202 were complete responses, meaning they filled all the questions to our survey and 169 were partial or incomplete responses, meaning that questions were skipped and left blank. In many cases, especially with our demographic information, we had 40% or more missing information from respondents. Information regarding how respondents viewed sharing the four interim result measures had less missing data, most likely because these were questions situated at the beginning of the survey. A potential reason for the amount of missing data may partially be that some people who are involved in trials may not be a part of generating or reviewing interim results, or regularly interacting with DSMBs or SCs and were thus less likely to be familiar with interim result measures. Nevertheless, it was important to include those interested or involved in trials as part of the sampling frame to capture the community’s understanding of which interim result measures should or should not be shared. On the contrary, it is possible that those that have experience being on, or interacting with, DSMBs are more acquainted with interim result measures and were thus more likely to answer the survey questions asked at the beginning that were related to these measures.

Another limitation is the possibility that an individual who is a member of both SCT and ISCB may have filled out the survey twice. We made a respondent’s anonymity and privacy a top priority and did not collect identifiable information that would allow us to crosscheck who filled out the survey from both societies. We also had to have a generic link to the survey because the survey was sent on our behalf by both SCT and ISCB. Thus, we could not provide a special and identifiable link to each unique respondent. However, if an individual was a member of both societies, it is possible that they remembered filling out the survey and would not elect to fill it out again as the same survey was used. The survey title page would have been recognizable before clicking the next button to officially start the survey. Additionally, it is important to note that most of our respondents, as indicated in Table 2, were statisticians and methodologists. In the future, it may be important to ensure a more balanced group of respondents to any survey related to this topic and make an additional effort to target non-statisticians/methodologists about these interim result measures. Their representation and interpretation on sharing these measures are equally important to understanding what interim result measures should be shared by the DSMB.

### Implications for practice

Trials are susceptible to bias and it is important to have a protocol with safeguards in place to prevent the introduction of biases that could alter trial results away from the true effect size, especially in phase III trials used to generate definitive results on efficacy and safety endpoints and provide evidence for practice and regulatory approval. In cases where there may be a request from non-DSMB members to have interim results shared with them by the trial’s DSMB, we do not recommend sharing the ICombinedER, IControlER, ACP or the UCP. Though there may be solid a priori plans in place in the trial protocol or DSMB charter to share the ICombinedER, as this measure is not directly unmasking of interim results on its own as the majority of respondents indicate, we think it lends non-DSMB members to make speculations about group rates, especially if there is a good inkling of the control rate in the academic literature. As mentioned before, the ICombinedER can also be interpreted to mean any one of three relative effects between groups making it a flawed measure to share and also lends non-DSMB members to mistakenly speculate on group rates. The results of this survey suggest that respondents from the trial community are not aware of this flaw with sharing the ICombinedER and may need to be educated on this issue. We should keep in mind that the DSMB needs to be trusted stewards of the trial and should be using discretion if there comes a time when sharing any of these four measures is needed. If such information had to be shared with a particular non-DSMB member, safeguards should be in place to prevent other non-DSMB members directly responsible for the operation or conduct of the trial, or those participating in the trial in some way, from knowing such interim result measures.

## Conclusion

From this survey, we have some empirical evidence that indicates that the IControlER, the ACP and the UCP should not be shared. Even though the majority of respondents indicated that ICombinedER could be shared, we do not recommend doing so. The ICombinedER can be unmasking of group rates if the IControlER is well known, either through the academic literature or some other source and also allows for mistaken speculation of groups rates as this measure can be interpreted in any of three ways; one group is performing better, worse or the same as the comparator group. The IControlER can be unmasking of group rates and the ACP is unmasking of relative treatment effects at interim. The UCP, on the other hand, is a confusing measure most likely because the measure is unfamiliar. With sharing any of these measures, there is a danger of introducing trial bias by non-DSMB members as it could alter their behavior towards the trial, consciously or subconsciously.

## Additional file

Additional file 1: Survey questions. (PDF 173 kb)

## Abbreviations

ACP: Adaptive Conditional Power
DSMB: Data Safety Monitoring Board
HiREB: Hamilton Integrated Research Ethics Board
ICombinedER: Interim Combined Event Rate
IControlER: Interim Control Event Rate
ISCB: International Society of Clinical Biostatistics
PI: Principle investigator
RCT: Randomized controlled trial
SC: Steering Committee
SCT: Society of Clinical Trials
UCP: Unconditional Conditional Power
