# Supplementary material for: Survey of professional views on sharing interim results by the Data Safety Monitoring Board (DSMB): what to share, with whom and why
Source: Trials. 2018 May 21;19:281. doi: 10.1186/s13063-018-2655-y (PMC5963112; doi:10.1186/s13063-018-2655-y)
Supplement: Supplementary file 1 — Survey questions. (PDF 173 kb) [file 13063_2018_2655_MOESM1_ESM.pdf]

Additional file 1: Survey questions

Question 1

Interim Combined Event Rate

"Interim Combined Event Rate is defined as:<br>*The total number of events observed at some planned interim point into the trial divided by the total number of participants admitted at that same planned interim point (e.g. a planned interim point can be six months from the start of the trial or after enrolling a certain number of participants).*<br><br>*Example:*<br><br>• *Total # of Deaths in both the placebo group and new intervention group, six months from the start of the trial = 80*<br>• *Total # of Participants in both the placebo group and the new intervention group, six months from the start of the trial = 700*<br>• *Calculation: 80/700 = 0.114 or 11.4%*<br>• *Therefore the Interim Combined Event Rate at the trial's interim analysis, six months from the start of the trial, is 11.4%" [1]*

1. Part A
During an ongoing Randomized Controlled Trial (RCT), do you think that the Data Safety Monitoring Board (DSMB) for an RCT should share the Interim Combined Event Rate with ANY of the following parties?

*(Please note that in most cases the sponsor is also the funder of the trial. By funder we are only referring to the entity which provides financial resources for the trial)

Please select ALL that apply.

A. Sponsor
B. The Steering Committee
C. The Investigator(s)
D. The Funder(s)*
E. Other, Please Specify ___________________
F. None of the Above

Advanced Branching: If answered A, B, C, D or E, show 1. Part B and 1. Part C. If answered F, show 1. Part D

1. Part B
How useful is it to share the Interim Combined Event rate at interim?

0<br>Not Useful at All	1	2	3	4	5	6	7	8	9	10<br>Very Useful

1. Part C
Please briefly explain why the Interim Combined Event Rates should be shared by the DSMB at interim.

1. Part D
Please briefly explain why the Interim Combined Event Rates should not be shared with anyone or any party at a trial's interim.

Question 2

Interim Control Event Rate

"Interim Control Event Rate is defined as:<br>*The number of events observed among control participants at some planned interim point into the trial divided by number of control participants admitted at that same planned interim point (e.g. a planned interim point can be six months from the start of the trial)*<br><br>*Example:*<br><br>• *Total # of Deaths in the placebo group, six months from the start of the trial = 15*<br>• *Total # of Participants in the placebo group, six months from the start of the trial = 250*<br>• *Calculation: 15/250 = 0.06 or 6%*<br>• *Therefore the Interim Control Event Rate at the trial's interim analysis, six months from the start of the trial, is 6%" [1]*

2. Part A
During an ongoing Randomized Controlled Trial (RCT), do you think that the Data Safety Monitoring Board (DSMB) for an RCT should share the Interim Control Event Rate with ANY of the following parties?

*(Please note that in most cases the sponsor is also the funder of the trial. By funder we are only referring to the entity which provides financial resources for the trial)

Please select ALL that apply.

A. Sponsor
B. The Steering Committee
C. The Investigator(s)
D. The Funder(s)*
E. Other, Please Specify ___________________
F. None of the Above

Advanced Branching: If answered A, B, C, D or E, show 2. Part B and 2. Part C. If answered F, show 2. Part D

2. Part B
How useful is it to share the Interim Control Event rate at interim?

0<br>Not Useful at All	1	2	3	4	5	6	7	8	9	10<br>Very Useful

2. Part C
Please briefly explain why the Interim Control Event Rates should be shared by the DSMB at interim.

2. Part D
Please briefly explain why the Interim Control Event Rates should not be shared with anyone or any party at a trial's interim.

Question 3

Adaptive Conditional Power

"Adaptive Conditional Power is defined as:
The probability of rejecting the null hypothesis of no effect by the end of the trial (i.e. finding a statistically significant effect in favour of the intervention), at some predetermined interim point in the trial when the adaptive conditional power is scheduled to be calculated. The assumption made is that the observed interim effect (i.e. relative risk reduction) in the trial will remain the same until the end of the trial.

Example statement:

Given the interim data (data collected 2 years into the trial that is planned to last for 3 years), and assuming the observed interim effect (i.e. relative risk reduction) at the two year mark to be the true effect for the remainder of the trial, the probability of rejecting the null hypothesis of no effect (i.e. finding a statistically significant effect in favour of the intervention) at the end of the trial is 60%.

The following pieces of information are used to calculate Adaptive Conditional Power at trial interim:

- *Control event rate and experimental event rate*
- *Information Fraction; a ratio of the planned sample size and the number of patients recruited in trial at the interim analysis*
- *Z score and B value at interim*
- *Drift parameter" [1]*

3. Part A
During an ongoing Randomized Controlled Trial (RCT), do you think that the Data Safety Monitoring Board (DSMB) for an RCT should share the Adaptive Conditional Power with ANY of the following parties?

*(Please note that in most cases the sponsor is also the funder of the trial. By funder we are only referring to the entity which provides financial resources for the trial)

Please select ALL that apply.

A. Sponsor
B. The Steering Committee
C. The Investigator(s)
D. The Funder(s)*
E. Other, Please Specify ___________________
F. None of the Above

Advanced Branching: If answered A, B, C, D or E, show 3. Part B and 3. Part C. If answered F, show 3. Part D

3. Part B
How useful is it to share the Adaptive Conditional Power at interim?

0<br>Not Useful at All	1	2	3	4	5	6	7	8	9	10<br>Very Useful

3. Part C
Please briefly explain why the Adaptive Conditional Power should be shared by the DSMB at interim.

3. Part D
Please briefly explain why the Adaptive Conditional Power should not be shared with anyone or any party at a trial's interim.

Question 4

Unconditional Conditional Power

"Unconditional Conditional Power is defined as:
The probability of correctly rejecting the null hypothesis of no effect at the end of the trial (i.e. finding a statistically significant effect in favour of the intervention) and accepting the alternative hypothesis when indeed the alternative hypothesis is true, at some interim point in the trial.

The following pieces of information are used to calculate Unconditional Conditional Power at interim:

- *The hypothesized treatment effect at the design stage (i.e. relative risk reduction) of the trial, assuming the hypothesized treatment effect at the design stage to be true and correct for the remainder of the trial;*
- *The sample size calculated at the design stage for the trial AND;*
- *The combined event rate calculated at the trial's interim, assuming this rate to be true for the remainder of the trial.*

Example statement:

Given the interim combined event rate and assuming the treatment effect (i.e. relative risk reduction) hypothesized at the design stage of the trial to be true for the remainder of the trial, the probability of correctly rejecting the null hypothesis of no effect (i.e. finding a statistically significant effect in favour of the intervention) at the end of the trial is 89%." [1]

4. Part A
During an ongoing Randomized Controlled Trial (RCT), do you think that the Data Safety Monitoring Board (DSMB) for an RCT should share the Unconditional Conditional Power with ANY of the following parties?

*(Please note that in most cases the sponsor is also the funder of the trial. By funder we are only referring to the entity which provides financial resources for the trial)

Please select ALL that apply.

A. Sponsor
B. The Steering Committee
C. The Investigator(s)
D. The Funder(s)*
E. Other, Please Specify ___________________
F. None of the Above

Advanced Branching: If answered A, B, C, D or E, show 4. Part B and 4. Part C. If answered F, show 4. Part D

4. Part B
How useful is it to share the Unconditional Conditional Power at interim?

0<br>Not Useful at All	1	2	3	4	5	6	7	8	9	10<br>Very Useful

4. Part C
Please briefly explain why the Unconditional Conditional Power should be shared by the DSMB at interim.

4. Part D
Please briefly explain why the Unconditional Conditional Power should not be shared with anyone or any party at a trial's interim.

Question 5

5. Part A
Do you think any other information should be shared during the interim of a Randomized Controlled Trial by the Data Safety Monitoring Board (DSMB)?

- ❑ **Yes**
- ❑ **No**

Advanced Branching: If answered yes, show 5. Part B

5. Part B
If yes, please briefly indicate what other information the DSMB should share at a trial's interim, with whom it should be shared, why, and how useful it is to share that information.
(You have the option of adding up to 5 different items)

What should be shared?	With whom should that information be shared?	Why should this information be shared?	From a scale between 0 to 10 (Where 0 is "Not Useful at All" and 10 is "Very Useful"), how useful is it to provide this information?
1.			0 1 2 3 4 5 6 7 8 9 10
2.			0 1 2 3 4 5 6 7 8 9 10
3.			0 1 2 3 4 5 6 7 8 9 10
4.			0 1 2 3 4 5 6 7 8 9 10
5.			0 1 2 3 4 5 6 7 8 9 10

Question 6

6. Part A
Have you ever been involved in a trial where it was explicitly stated in the Data Safety Monitoring Board (DSMB) charter *what interim information/data/results should be shared* AND *with whom that information should be shared* during the trial's interim?

- ❑ **Yes**
- ❑ **No**

Advanced Branching: If answered yes, show 6. Part B, 6. Part C and 6. Part D

6. Part B
According to any DSMB charter(s) you encountered, please indicate below whether any of the following pieces of interim information *should be shared* during the interim of a trial, *with whom* and *under what circumstance* the sharing would happen, where applicable.

(*If you would like to see the definitions of the interim pieces of information in this chart below, please select "yes" to the checkbox at the bottom of this page and the definitions will appear below. Deselect "Yes" if you want the definitions to disappear)

**Interim Information**	**Please select if any of the following pieces of interim information should be shared according to any DSMB charter(s) you encountered.**	**With whom should this interim information be shared? (e.g. Investigator(s), Sponsor, Steering Committee, Funder of the Trial, etc.)**	**Under what circumstance(s) should this interim information be shared?**
Interim Combined Event Rate	❑		
Interim Control Event Rate	❑		
Adaptive Conditional Power	❑		
Unconditional Conditional Power	❑		

6. Part C
According to any DSMB charter(s) you encountered, *should any other information* be shared during the interim of a trial, *with whom* and *under what circumstance*, where applicable.

(You have the option of adding up to 5 different items if you would like)

**Interim Information that should be shared**	**With whom should this interim information be shared? (e.g. Investigator(s), Sponsor, Steering Committee, Funder of the**	**Under what circumstance(s) should this interim information be shared?**
1.		
2.		
3.		
4.		
5.		

6. Part D
***If you would like to see the definitions for the first chart above, please select "Yes" below. (To make the definitions disappear, deselect "Yes" below)**

- ❑ **Yes**

Advanced Branching: If answered yes, show 6. Part E

6. Part E
Definitions for Reference

____

***"Interim Combined Event Rate* is defined as:**
The total number of events observed at some planned interim point into the trial divided by the total number of participants admitted at that same planned interim point (e.g. a planned interim point can be six months from the start of the trial or after enrolling a certain number of participants).

Example:

- ***Total # of Deaths in both the placebo group and new intervention group, six months from the start of the trial = 80***
- ***Total # of Participants in both the placebo group and the new intervention group, six months from the start of the trial = 700***
- ***Calculation: 80/700 = 0.114 or 11.4%***
- ***Therefore the Interim Combined Event Rate at the trial's interim analysis, six months from the start of the trial, is 11.4%" [1]***

____

"Interim Control Event Rate is defined as:
The number of events observed among control participants at some planned interim point into the trial divided by number of control participants admitted at that same planned interim point (e.g. a planned interim point can be six months from the start of the trial)

Example:

- ***Total # of Deaths in the placebo group,*** **six months from the start of the trial =** ***15***
- ***Total # of Participants in the placebo group,*** **six months from the start of the trial =** ***250***
- ***Calculation: 15/250 = 0.06 or 6%***
- ***Therefore the Interim Control Event Rate at the trial's interim analysis,*** **six months from the start of the trial,** ***is 6%" [1]***

____

"Adaptive Conditional Power is defined as:
The probability of rejecting the null hypothesis of no effect by the end of the trial (i.e. finding a statistically significant effect in favour of the intervention), at some predetermined interim point in the trial when the adaptive conditional power is scheduled to be calculated. The assumption made is that the observed interim effect (i.e. relative risk reduction) in the trial will remain the same until the end of the trial.

Example statement:

- *Given the interim data (data collected 2 years into the trial that is planned to last for 3 years), and assuming the observed interim effect (i.e. relative risk reduction) at the two year mark to be the true effect for the remainder of the trial, the probability of rejecting the null hypothesis of no effect (i.e. finding a statistically significant effect in favour of the intervention) at the end of the trial is 60%.*

The following pieces of information are used to calculate Adaptive Conditional Power at trial interim:

- *Control event rate and experimental event rate*
- *Information Fraction; a ratio of the planned sample size and the number of patients recruited in trial at the interim analysis*
- *Z score and B value at interim*
- *Drift parameter" [1]*

____

"Unconditional Conditional Power is defined as:
The probability of correctly rejecting the null hypothesis of no effect at the end of the trial (i.e. finding a statistically significant effect in favour of the intervention) and accepting the alternative hypothesis when indeed the alternative hypothesis is true, at some interim point in the trial.

The following pieces of information are used to calculate Unconditional Conditional Power at interim:

1. **The <u>hypothesized treatment effect at the design stage</u> (i.e. relative risk reduction) of the trial, assuming the hypothesized treatment effect at the design stage to be true and correct for the remainder of the trial;**
2. **The <u>sample size calculated at the design stage</u> for the trial AND;**
3. **The <u>combined event rate calculated at the trial's interim</u>, assuming this rate to be true for the remainder of the trial.**

Example statement:

- *Given the interim combined event rate and assuming the treatment effect (i.e. relative risk reduction) hypothesized at the design stage of the trial to be true for the remainder of the trial, the probability of correctly rejecting the null hypothesis of no effect (i.e. finding a statistically significant effect in favour of the intervention) at the end of the trial is 89%". [1]*

Question 7
Please identify if you are currently or have been in one of these roles in relation to the operation of a trial.

Please select ALL that apply:

☐	A. Trialist or Investigator (i.e. Co-Investigator in a trial)
☐	B. Principal Investigator (PI) of a clinical trial or RCT
☐	C. Data Safety Monitoring Board (DSMB) Member
☐	D. Research Nurse
☐	E. Trial Coordinator
☐	F. Representative of the sponsor of the trial (The sponsor of the trial is responsible for trial initiation, administration and management. In many case they also help to fund/finance the trial)
☐	G. Representative of the funder** of the trial (**Please note that in most cases the sponsor is also the funder of the trial. By funder we are only referring to the entity which provides financial resources for the project)
☐	H. Trial statistician
☐	I. Data Analyst
☐	J. Data Manager
☐	K. Other role within a trial, Please describe: ______________________

Question 8
How many trials have you been involved with?

Please select ONLY ONE:

A. None
B. 1 to 5 trials
C. 6 to 10 trials
D. 11 to 15 trials
E. More than 15 trials

Question 9
What do you regard as your primary or main profession by training?

Please select ONLY ONE:

A. Physician
B. Nurse or Nurse Practitioner
C. Pharmacist
D. Dentist
E. Methodological Scientist/Research Methodologist
F. Epidemiologist
G. Medical Laboratory Scientist
H. Physiotherapist
I. Occupational Therapist
J. Optometrist
K. Psychologist
L. Midwife
M. Ethics specialist
N. Lawyer
O. Research or Clinical Trial Coordinator
P. Medical Laboratory Technician
Q. Mathematician/Statistician
R. Computer Scientist
S. Information Technologist
T. Computer Programmer
U. Data Manager
V. Other Profession, Please Describe: ______________________

Question 10

What other professional roles have you taken on? (Please exclude the option selected in Question 9)

Please select ALL that apply:

☐	A. Physician
☐	B. Nurse or Nurse Practitioner
☐	C. Pharmacist
☐	D. Dentist
☐	E. Methodological Scientist/Research Methodologist
☐	F. Epidemiologist
☐	G. Medical Laboratory Scientist
☐	H. Physiotherapist
☐	I. Occupational Therapist
☐	J. Optometrist
☐	K. Psychologist
☐	L. Midwife
☐	M. Ethics specialist
☐	N. Lawyer
☐	O. Research or Clinical Trial Coordinator
☐	P. Medical Laboratory Technician
☐	Q. Mathematician/Statistician
☐	R. Computer Scientist
☐	S. Information Technologist
☐	T. Computer Programmer
☐	U. Data Manager
☐	V. Other Profession, Please Describe: ______________________

Question 11

In what setting or environment do you usually work?

Please select ONLY ONE:

A. Hospital
B. Medical or Health Clinic
C. Private Practice
D. University or Academic Institution
E. Government Research Group
F. Government Regulatory Body
G. Private or Contracted Research Company
H. Pharmaceutical Company
I. Medical Device Company
J. Other. If so, please indicate, in general, the setting you usually work? ______________________

Question 12

In what other settings or environments do you usually work? (Please exclude the option selected in Question 11)

Please select ALL that apply:

☐	A. Hospital
☐	B. Medical or Health Clinic
☐	C. Private Practice
☐	D. University or Academic Institution
☐	E. Government Research Group
☐	F. Government Regulatory Body
☐	G. Private or Contracted Research Company
☐	H. Pharmaceutical Company
☐	I. Medical Device Company
☐	J. Other. If so, please indicate, in general, the setting you usually work? ______________________

Question 13
How many of the trials that you have been involved with had some form of private industry sponsorship?

Please select ONLY ONE:

A. None
B. 1 to 5 trials
C. 6 to 10 trials
D. 11 to 15 trials
E. More than 15 trials

Question 14
How many of the trials that you have been involved with had a Data Safety Monitoring Board (DSMB) monitoring the trial?

Please select ONLY ONE:

A. None
B. 1 to 5 trials
C. 6 to 10 trials
D. 11 to 15 trials
E. More than 15 trials

Reference

[1] V. Borg Debono, L. Mbuagbaw, J. Paul, N. Buckley, L. Thabane, Sharing some interim data in trial monitoring can mislead or unmask trial investigators: A scenario-based survey of trial experts, Contemporary Clinical Trials Communications 7 (2017) 81-85.
